# Internal Fixation Using Vitcone^®^ Locking-Plate System for Fractures of Radius and Ulna in Toy- and Small-Breed Dogs: A Retrospective Two-Center Clinical Study

**DOI:** 10.3390/ani16182928

**Published:** 2026-09-17

**Authors:** Andrea Urizzi, Umberto Maggiolini, Andrea Paolini, Francesco Collivignarelli, Roberto Tamburro

**Affiliations:** 1 Clinca Veterinaria Urizzi, 30028 San Michele al Tagliamento, Italy; andrea@urizzi.com; 2 Department of Veterinary Medicine, University of Teramo, 64100 Teramo, Italy; umaggiolini@unite.it; 3 Department of Life Science and Health Profession, Link University, 00165 Rome, Italy; f.collivignarelli@unilink.it

**Keywords:** radius, ulna, fracture, Vitcone^®^ locking system

## Abstract

This retrospective two-center study describes clinical and radiographic outcomes in dogs weighing up to 10 kg undergoing surgical stabilization of radius–ulna fractures using either the Vitcone^®^ locking system (Group A) or the Veterinary Instrumentation (Vi^®^) locking system (Group B) between January of 2021 and December of 2025. Fractures were stabilized with open reduction and internal fixation using a cranio-medial or cranio-lateral approach. Ninety-three dogs met the inclusion criteria (59 in Group A, 34 in Group B). In Group A, the cranio-lateral approach was used in 57% of cases and the cranio-medial approach in 43%, whereas Group B relied exclusively on the cranio-medial approach. At the 12-week recheck, radiographic healing was assessed by two independent observers using a Visual Assessment Scale (VAS), with intraclass correlation coefficients (ICCs) > 0.75 in both groups. Implant removal was performed following the surgeon’s assessment in 68% of dogs in Group A and 36% in Group B. Major complications occurred in 8.4% of dogs in Group A, with one implant breakage and four refractures following implant removal, and three refractures occurred in Group B. Long-term owner follow-up, when available, indicated satisfactory limb use in daily activities. This study provides clinically useful observational data on the application of Vitcone^®^ and Vi^®^ locking systems for stabilizing radius–ulna fractures in small-breed dogs, suggesting their role as viable options in routine surgical practice.

## Simple Summary

Radius and ulna fractures in small-breed dogs are common orthopedic injuries, the management of which is often challenging due to the unique anatomical, biomechanical, and vascular characteristics of the distal antebrachium in these patients. This study describes the clinical outcomes of 93 dogs with radius–ulna fracture treated with two different locking-plate systems: the Vitcone^®^ locking system (59/93) and the Veterinary Instrumentation^®^ locking system (34/93). At follow-up, most dogs showed subjective clinical improvement, with absence of observable lameness on clinical examination when available. Owner telephone reports indicated satisfactory limb use in daily activities, although these assessments were not based on a validated functional outcome measure. Major complications included refracture after implant removal in both groups and one plate breakage in group A. These findings provide a descriptive overview of how both fixation systems performed in clinical practice and highlight the importance of careful implant removal planning in young, small-breed dogs.

## 1. Introduction

Fractures of the radius and ulna are common orthopedic injuries in canine patients, representing approximately 8.5 to 17% of all fractures [1,2,3,4]. In dogs weighing less than 10 kg, the distal antebrachium is characterized by low vascular blood supply, making it highly susceptible to vascular compromise following trauma [5,6]. In addition, toy-breed dogs have a distal radius and ulna with smaller cross-sectional areas, reduced medullary cavities, lower moments of inertia, and proportionally thicker cortices—a geometric configuration that makes their forearm bones biomechanically weaker and more prone to fracture than those of medium- and large-sized dogs [4]. These fractures tend to be transverse or short-oblique and are notorious for progressing to non-union if not managed with surgical fixation [7,8]. These anatomical conditions may predispose these dogs to delayed union, non-union, and implant failure when stabilization is inadequate or biological support is insufficient [9].

Small-breed dogs, particularly Chihuahuas, Pomeranians, Toy Poodles, and Yorkshire Terriers, exhibit a well-documented predisposition to distal radial fractures, often resulting from low-energy trauma [4,7].

A variety of treatment modalities have been employed for radial and ulnar fractures, ranging from surgical stabilization using external skeletal fixation to dynamic compression plates and, more recently, steel and titanium locking-plate technology [8,10,11,12,13,14,15,16].

The introduction of locking compression plates (LCPs) has represented a major advancement in the management of antebrachial fractures. Locking constructs provide angular stability, reduce the need for precise plate-to-bone contouring, and preserve periosteal blood supply by minimizing plate–bone contact [17].

These features are particularly advantageous in small-breed dogs, where biological fragility and limited bone stock increase the risk of fixation failure [18]. Several studies have demonstrated improved outcomes with locking plates compared with traditional non-locking constructs, including higher union rates, reduced complication rates, and better maintenance of alignment [10,11,13,19,20,21]. Both open reduction and minimally invasive approaches have been described in previous studies [22,23,24]. However, the occurrence of major postoperative complications requiring revision surgery for fracture treatment using conventional and locking-plate systems have been reported as 3% and 9%, respectively [23,25]. Nevertheless, the optimal fixation strategy remains a topic of debate. One study has reported that proper reduction of the caudal cortex of the radius is critical to promote bone healing, thus preventing implant failure [26].

Given the complexity of these injuries and the diversity of clinical presentations, there is a continued need for high-quality research that clarifies optimal treatment strategies and identifies predictors of successful outcomes.

**Figure 1:**
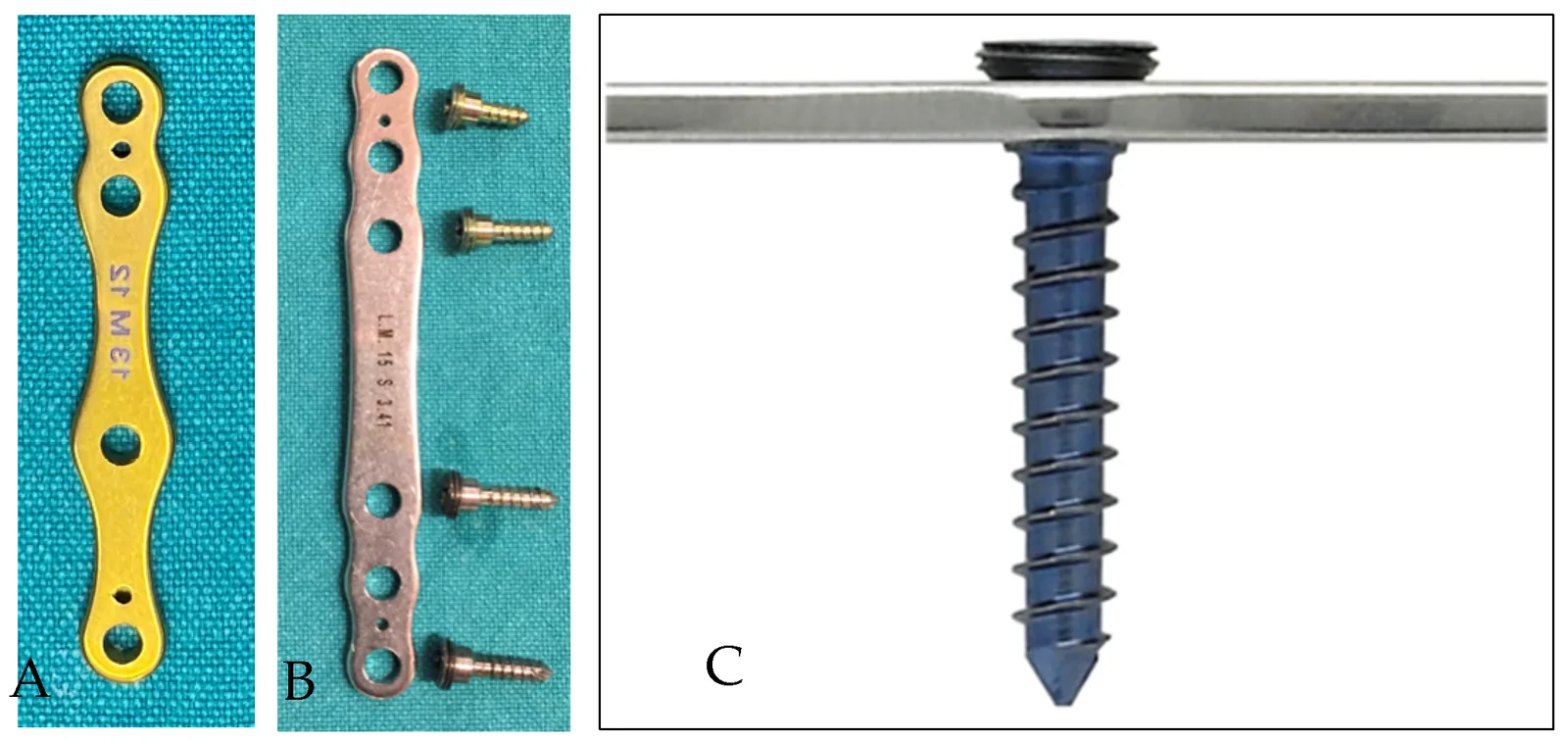
Vitcone® locking system. Plate 13-M-12: length 35.6 mm, thickness 1.3 mm, holes n. 4 (**A**); plate 15-S-3.41: length 65 mm, thickness 1.5 mm, holes n. 6 with 2.5 mm screws (**B**). Conical locking occurs between the non-protruding portion of the screw head and the conical hole in the plate; part of the screw head protrudes above the plate (**C**).

The present study aims to report outcomes and complication rates in dogs undergoing radial and ulnar fractures stabilized using two internal fixation system: the Vitcone^®^ internal fixation system (Domeggedi Cadore, Belluno, Italy) and the Veterinary Instrumentation (Vi^®^) locking system (Veterinary Instrumentation, Sheffield, UK). The Vitcone^®^ locking-plate system is an AISI 316LVM stainless steel or Ti-6Al-4V titanium alloy locking-plate system that achieves angular stability through direct conical coupling between the plate and screw heads. The self-tapping and self-locking screws are produced in Ti-6Al-4V titanium alloy, although they can also be made from AISI 316LVM steel (Figure 1). No research thus far has described the application of the internal fixator system for management of radio-ulnar fractures in small-breed dogs. We therefore aimed to describe the clinical and radiographic outcomes and complication patterns associated with the Vitcone^®^ locking system in small-breed dogs.

## 2. Materials and Methods

This descriptive retrospective study evaluated dogs weighing up to 10 kg undergoing surgical stabilization of radius–ulna fractures using the Vitcone^®^ locking system (Domegge di Cadore, Belluno, Italy) and the Vi^®^ internal locking system (Veterinary Instrumentation, Sheffield, UK) between January of 2021 and December of 2025.

All cases were enrolled consecutively, and both fixation systems were used during overlapping time intervals within the study period.

Informed written owner consent was obtained for all dogs enrolled in this study, permitting the use of their clinical data for research purposes, in accordance with the routine policy of the Veterinary Teaching Hospital of the University of Teramo. Dogs were eligible for inclusion if they presented with a complete diaphyseal or metaphyseal fracture of the radius and ulna and had no concurrent neurological conditions affecting the same limb. Exclusion criteria included open fractures, pathological fractures secondary to neoplasia or metabolic disease, incomplete medical records, or lack of postoperative radiographic follow-up.

All dogs were divided into two groups based on the locking system used for fracture stabilization: Group A (Vitcone^®^ locking system) and Group B (Vi^®^ locking system). The Vitcone^®^ system consists of a low-profile locking plate with fixed-angle screws. The Vi^®^ locking system consists of stainless-steel plates with threaded screw holes allowing angular-stable fixation. Both implants were applied according to manufacturer guidelines, and the choice of system depended on surgeon preference and implant availability.

All Group A cases were performed by A.U., and all Group B cases by R.T. As each fixation system was used exclusively at one institution by a single surgeon, fixation system, center, and surgeon were completely confounded and inseparable. The Veterinary Instruments cohort was not intended as a control group; its inclusion provides descriptive real-world data from a second institution.

For each dog, the clinical history, signalment, body weight, and the time elapsed from trauma to presentation were reviewed and recorded.

Preoperatively, orthopedic examination was performed in all dogs. Lameness was subjectively assessed based on a five-grade scoring system as follows: 0/4 no lameness; 1/4 slight; 2/4 mild; 3/4 moderate; and 4/4 no weight-bearing. Medio-lateral and cranio-caudal preoperative radiographs of the affected forelimb were performed, and fractures were classified according to the AO fracture classification system by two independent observers (AU, UM). All data were retrospectively reviewed and analyzed.

Dogs were premedicated with methadone (Confortane, Dechra Veterinary Products, Northwich, Cheshire, UK), 0.2 mg/kg IM, and dexmedetomidine (Dexdomitor, Zoetis, Parsippany, NJ, USA), 5 mcg/kg IM. Then, anesthetic induction for endotracheal intubation was performed using propofol (PropoVet, Fatro, Bologna, Italy), titrated to effect and maintained on isoflurane in oxygen.

Each dog was positioned in lateral recumbency according to the selected cranio-medial or cranio-lateral surgical approach. Following skin and subcutaneous incision, soft tissue dissection was minimized to preserve periosteal blood supply. Fracture reduction was achieved via an open reduction–internal fixation approach followed by direct manipulation and pointed reduction forceps. In reducible fractures, anatomical reconstruction was attempted; in comminuted fractures, a bridging technique was used without reduction of intermediate fragments. Care was taken to avoid penetration of the radiocarpal joint and to maintain appropriate alignment in all planes. Implant size selection was based on patient size: in Group A, 2.5 mm plates were used in dogs weighing 5–10 kg and 1.8 mm plates in dogs < 5 kg; in Group B, 2.4 mm plates were used in dogs 5–10 kg and 2.0 mm plates in dogs < 5 kg.

Postoperatively, medio-lateral and cranio-caudal radio-ulnar radiographs were used to evaluate bone alignment, fracture line apposition, and apparatus. Postoperative management included a modified Robert Jones bandage for 2–3 days, restricted activity for 6 to 8 weeks with leash walks (5–10 min, three times daily), and progressive return to normal exercise thereafter. All dogs received antibiotic prophylaxis with Amoxicillin/Clavulanic Acid (Synulox, Fatro, Bologna, Italy), 20 mg/kg BID, for 7 days and the non-steroidal anti-inflammatory drug Meloxicam (Metacam, Boehringer, Ingelheim am Rhein, Germany), 0.1 mg/kg SID, for 10 days after surgery.

Follow-up clinical data and radiographs were obtained at approximately 8 or 12 weeks, or until complete healing was documented. Lameness was subjectively assessed based on the same five-grade scoring system (0–4) used preoperatively. Radiographic follow-up was performed at approximately 12 weeks postoperatively, depending on the scheduling availability of each institution. Radiographic healing was evaluated independently by two observers using a 0–4 ordinal Visual Analog Scale (VAS), where each category corresponds to a percentage range of cortical bridging, as follows: 0 = 0%, 1 = 1–25%, 2 = 26–50%, 3 = 51–75%, 4 = 76–100% [27]. Each radiographic projection (medio-lateral and cranio-caudal) was scored separately. VAS scores were not averaged, converted into continuous percentages, or combined into a dog-level mean value. The VAS was used exclusively to assess inter-observer agreement based on the intraclass correlation coefficient (ICC). VAS data were tested for normality using the Shapiro–Wilk test, and mean values and standard deviations were calculated for each observer. The ICC ranged from 0 (no agreement) to 1 (excellent agreement). Values < 0.40 were considered poor agreement, values between 0.40 and 0.59 fair agreement, values between 0.60 and 0.74 good agreement, and values ≥ 0.75 excellent agreement.

Complications were classified as minor or major according to the criteria described by Cook et al. [28]. Minor complications were managed conservatively, whereas major complications required surgical revision. Major complications were further categorized as implant-related failures or complications occurring after implant removal and were summarized descriptively. Fisher’s exact test (Jamovi 2.3) was calculated but not used for inferential interpretation, due to the very small number of events and the complete confounding between fixation system, center, and surgeon. Long-term follow-up was performed by phone call approximately one year after surgery, asking owners whether or not the patient was lame on the treated forelimb.

## 3. Results

Over the 5-year observation period, a total of 119 medical records were reviewed. Ninety-three cases of dogs with unilateral radius–ulna fractures who met the inclusion criteria (56 right forelimb; 37 left forelimb) were treated. Group A and Group B included 59/93 (63%) and 34/93 (37%) of cases, respectively (Table 1). Twenty-six cases were excluded due to open fractures (4/26) and incomplete records (15/26).

In Group A, the cranio-medial approach was used in 43% of dogs during the earlier cases, whereas the cranio-lateral approach was used in 57% of dogs as the surgeon progressively shifted their preference over time. In Group B, a cranio-medial approach was used in 100% of cases (Table 2). Plate selection is summarized in Table 2.

Limb function was subjectively assessed as satisfactory at the 12-week recheck in most dogs: 88% in Group A and 76% (26/34) in Group B showed a lameness score of 0/4. Range of motion (ROM) reduction in the radio-carpal joint occurred only in dogs with distal fractures, but the small number of events and the strong association between surgical approach and fixation system, institution, surgeon, and time period do not allow for any interpretation regarding the effect of the approach. The ROM findings are therefore presented descriptively.

**Figure 2:**
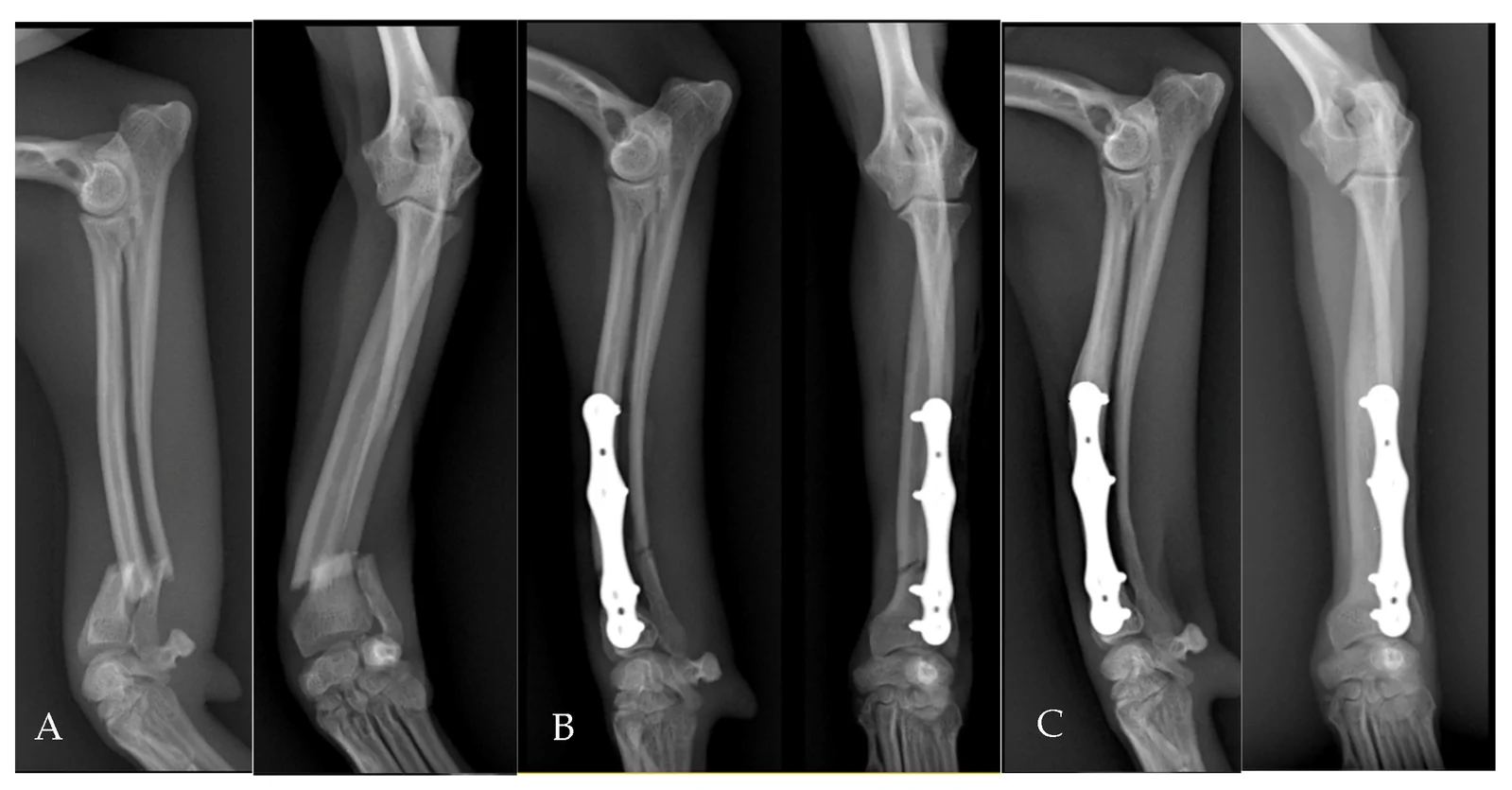
Group A: A one-year-old male Chihuahua dog, 1.9 kg, with distal radius and ulna fracture of the right forelimb treated with a lateral approach to the radius. Medio-lateral (ML) and cranio-caudal (Cr-Cd) preoperative (**A**), postoperative (**B**), and 3-month follow-up (**C**) radiographic views, showing evidence of complete healing. Implant length: 35.6 mm; thickness: 1.3 mm; holes n.: 4; VAS: 100%.

Radiographic progression of healing typically showed cortical bridging within 8–12 weeks in both groups. At the 8–12-week recheck, all dogs with available radiographs were assigned a VAS score of 4 (corresponding to 76–100% healing) on both projections by both observers. VAS scoring was used solely to assess inter-observer agreement (Figure 2).

Inter-observer agreement was excellent, with ICC values > 0.75 for both groups. These radiographic findings are descriptive and were not subjected to statistical comparison between groups (Table 3).

**Figure 3:**
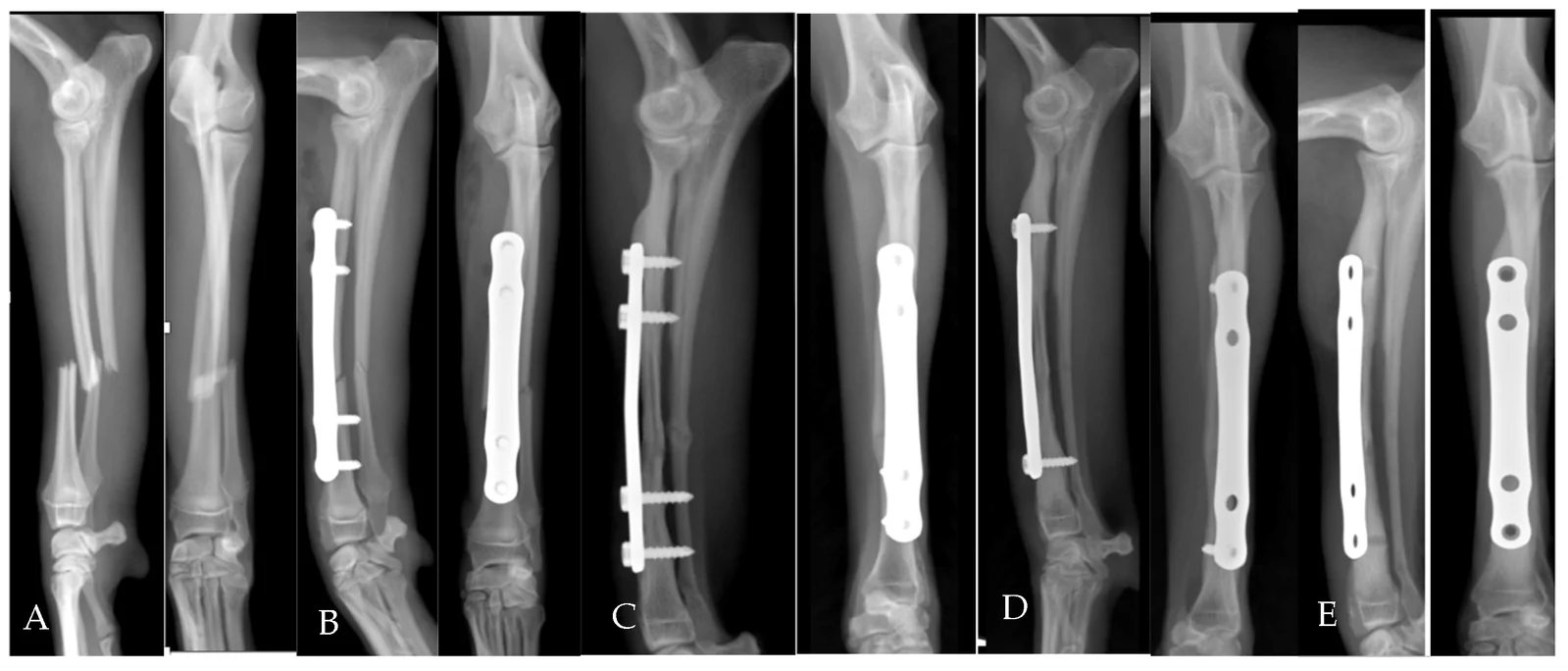
Group A: A 7-month-old male Poodle dog, 3.9 kg, with diaphyseal radius and ulna fracture of the left forelimb. ML and Cr-Cd preoperative (**A**) and postoperative (**B**) views. Two-month (**C**), four-month (**D**) and six-month (**E**) postoperative radiographic views. Two screws were progressively removed at the two- and four-month follow-ups, leading to partial implant removal (screw removal and plate retention).

Implant removal was performed only when clinically indicated (e.g., soft-tissue irritation, discomfort, owner request) or when stress protection was considered a potential risk in dogs with advanced healing (Figure 3), (Table 4).

**Figure 4:**
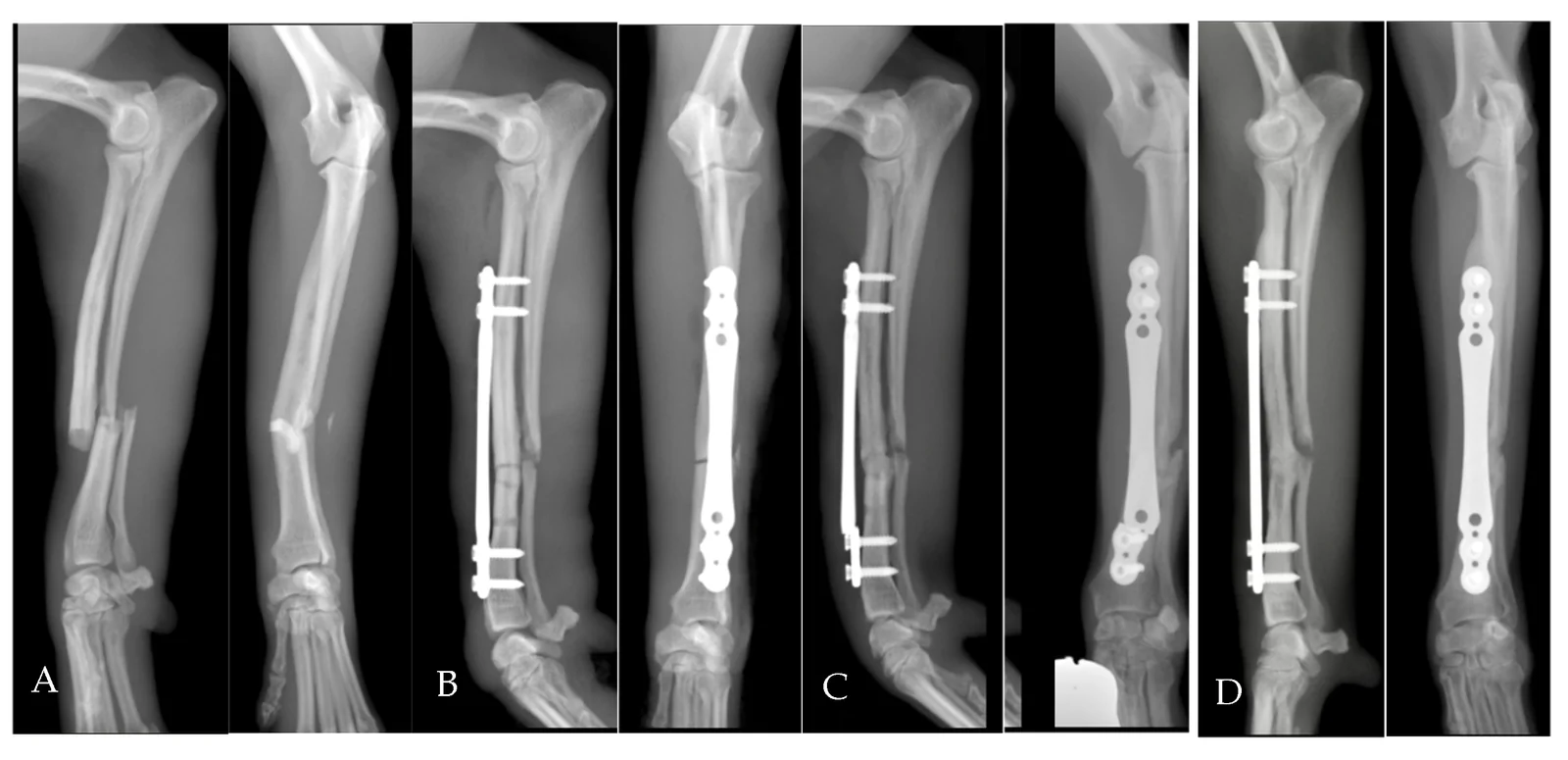
Group A: A 7-year-old female Pinscher dog, 3 kg, with diaphyseal radius and ulna fracture of the right forelimb. ML and Cr-Cd preoperative (**A**) and postoperative (**B**) views. Twenty-nine days after surgery, evidence of plate breakage was observed (**C**); two months after revision surgery using the same implant size, bone healing was achieved (**D**).

**Figure 5:**
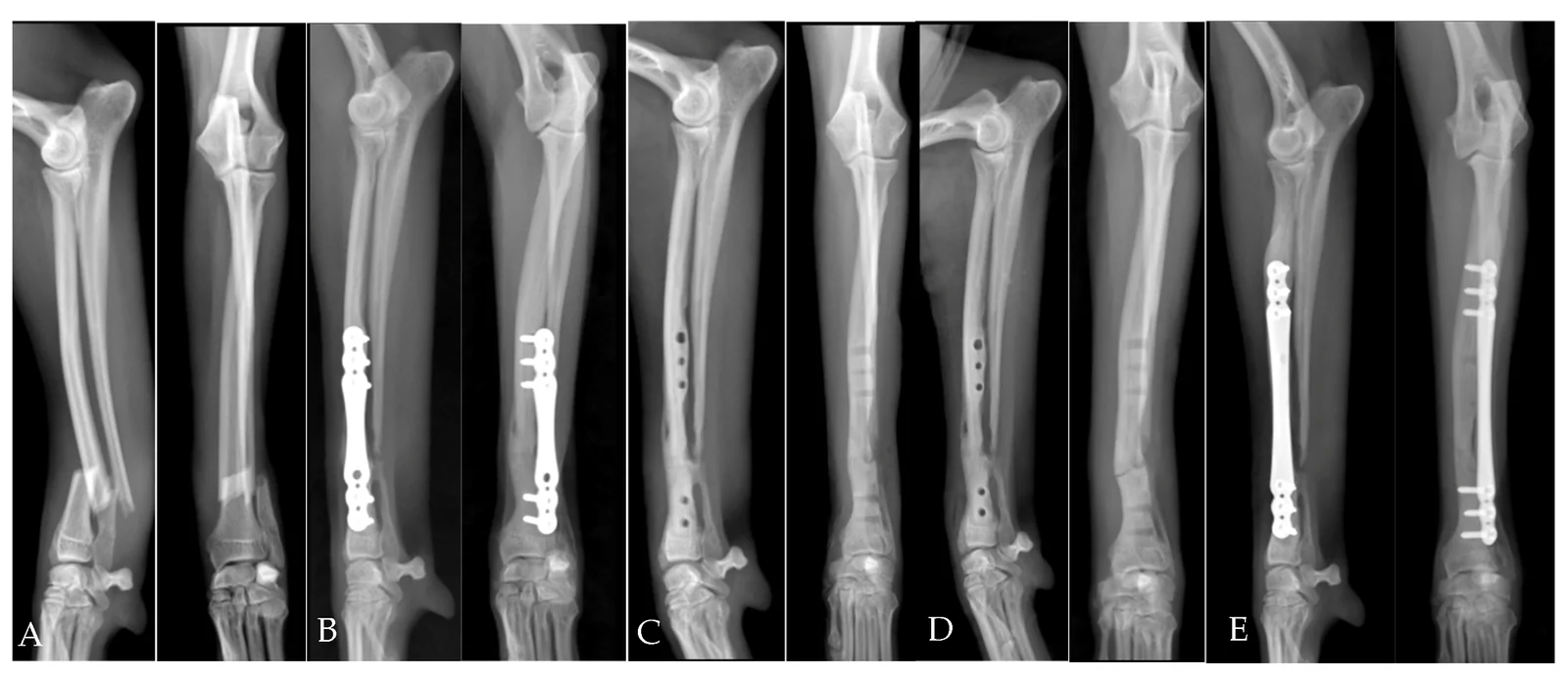
Group A: A 1-year-old male Poodle dog, 5 kg, with distal radius and ulna fracture of the left forelimb. ML and Cr-Cd preoperative (**A**) and postoperative (**B**) views. Implant removal was performed 8 weeks after surgery (**C**); evidence of refracture 17 days after implant removal due to low-energy trauma (**D**); revision surgery was performed (**E**).

The overall complication rate was 12% (11/93). Major complications occurred in 9% (8/93) and minor complications in 3% (3/93) of cases (Figure 4 and Figure 5), (Table 5).

Long-term follow-up was available for 80% (47/59) of dogs in Group A and 67.6% (23/34) in Group B. Owners reported full weight-bearing in all contacted cases.

## 4. Discussion

Radius–ulna fractures in small-breed dogs remain clinically challenging due to the unique anatomical and biomechanical characteristics of the distal antebrachium. The demographic distribution of the present cohort, dominated by toy- and small-breed dogs affected by low-energy trauma, aligns with previously published epidemiology [1,23]. This study provides descriptive information from two institutions using different locking-plate systems, offering insight into real-world variability in case presentation, surgical management, and postoperative progression. Because the fixation system, institution, surgeon, and clinical protocols were completely confounded, the two cohorts cannot be interpreted comparatively.

The findings therefore address the hypothesis by providing a descriptive overview of healing progression and complication patterns in small-breed dogs undergoing locking-plate fixation.

Surgical approaches differed between institutions, reflecting surgeon preference and evolving practice patterns. The cranio-medial approach has traditionally been recommended for radial fractures because it provides direct access to the tension surface of the radius [6]. Cadaveric studies have highlighted potential limitations related to soft-tissue handling, particularly the proximity to the extensor carpi radialis tendon and the restricted ability to retract surrounding structures safely [29,30]. The cranio-lateral approach has been proposed as an alternative corridor that may facilitate exposure while avoiding manipulation of the extensor carpi radialis tendon [29,30]. Although reduced radiocarpal range of motion was observed only in distal fractures, the present data cannot support any interpretation regarding the effect of surgical approach, as approach was inseparably linked to fixation system, institution, surgeon, and time period. Variability in ROM likely reflects case heterogeneity, postoperative factors, and the non-standardized nature of clinical assessment. Prospective studies with validated functional evaluation would be required to investigate this aspect.

Radiographic healing progressed within the timeframe commonly reported for stable internal fixation of radial fractures in small-breed dogs, with cortical bridging typically observed within eight weeks [6]. The Visual Assessment Scale (VAS) provided a practical, semiquantitative method for describing healing progression, and high inter-observer agreement (ICC > 0.75) supports the reliability of the radiographic assessment. However, the modified VAS used in this study was applied only to evaluate inter-observer consistency and does not provide validated information on sensitivity, responsiveness, or clinical reliability. The radiographic findings were therefore interpreted qualitatively, without comparative analysis between cohorts. The most clinically significant finding is that radiographic healing consistently progressed within eight weeks, which aligns with previously reported healing intervals for stable internal fixation in small-breed dogs.

Refractures occurred primarily after implant removal, consistent with previous reports indicating that early plate removal in immature dogs increases the risk of refracture due to incomplete remodeling and persistent cortical weakness [25]. A recent study identified reduced radial thickness at the fracture site as a key risk factor for refracture in toy-breed dogs following plate removal [31]. In the present cohort, implant removal practices varied between institutions and were not protocol-driven. In one institution, removal was frequently performed using minimally invasive techniques and motivated by concerns about stress shielding, whereas staged dynamization was more common in the other. These strategies represent institution-specific preferences rather than distinct clinical approaches and cannot be interpreted comparatively. Refracture after dynamization or screw removal should be considered a complication occurring after a secondary elective procedure, rather than a failure of the original fixation.

Clinically, these findings emphasize that stable locking-plate fixation enables predictable healing in small-breed dogs, whereas implant removal, particularly in immature patients, remains a critical decision point associated with increased refracture risk.

This study has several important limitations. Treatment allocation was determined entirely by institution-specific practice, resulting in complete confounding between fixation system, center, surgeon, and time period. The retrospective design, unequal group sizes, heterogeneous follow-up, unblinded outcome assessment, and non-validated clinical scoring further limit interpretability. The very low number of major complications, incomplete outcome data, and differential loss to follow-up preclude any causal or comparative interpretation. For these reasons, the findings should be considered descriptive of the clinical performance and complication patterns observed in this cohort. Prospective controlled studies with standardized protocols, validated outcome measures, and clearly defined implant removal criteria are required to further investigate these aspects and may contribute to the development of evidence-based guidelines for the management of radial fractures in small-breed dogs.

## 5. Conclusions

This retrospective study described the clinical use of the Vitcone locking system for radial fractures in small-breed dogs. Stable internal fixation resulted in predictable radiographic healing within eight weeks, consistent with previously reported healing intervals for similar dog populations. Refractures occurred primarily after implant removal, underscoring the importance of cautious removal timing, especially in immature patients. These findings provide clinically relevant descriptive information on healing progression and complication patterns, but do not allow for comparative interpretation due to complete confounding between fixation system, institution, and surgeon. Prospective controlled studies with standardized protocols and validated outcome measures are required to further clarify these aspects and support the evidence-based management of radial fractures in small-breed dogs.

## Figures and Tables

**Table 1:** Summary of case numbers, breed distribution, BW, age, sex, and affected side.

	Group A ^1^	Group B ^2^
- Case numbers	59/93 (63%)	34/93 (37%)
- Mixed breed	19/59 (32%)	17/34 (50%)
- Pinscher	11/59 (19%)	5/34 21%)
- Poodles	12/59 (20%)	7/34(15%)
- Yorkshire	6/59 (10%)	1/34 (3%)
- Chihuahua	3/59 (5%)	3/34 (9%)
- Pomeranian	4/59 (7%)	-
- Whippet	4/59/7%)	-
- Median age and range	3 yo (6 m–8 yo)	4 yo (1 yo–10 yo)
- Sex (M/F)	24 M; 35 F	20 M; 14 F
- Median BW and range	4 kg (1.8–9 kg)	4 kg (2.1–10 kg)
- R/Left side	43 R; 16 L	13 R; 21 L

^1^ Vitcone^®^ locking system; ^2^ Vi^®^ locking system; yo = years old, m = months old; BW = body weight.

**Table 2:** Summary of fracture localization, surgical approaches, and postoperative implant management.

	Group A ^1^	Group B ^2^
- Diaphyseal fracture	21/59 (36%)	10/34 (30%)
- Distal fracture	38/59 (64%)	24/34 (70%)
- Comminution	8/59 (13%)	2/34 (6%)
- Bone grafting	1/59 (1.7%)	0/34 (0%)
- Lateral approach	34/59 (57%)	-
- Medial approach	25/59 (43%)	34/34 (100%)
- Median time to surgery and range	2 days (1–5 days)	3 days (1–6 days)
- 2.5 mm plate	14/59 (24%)	-
- 1.8 mm plate	45/59 (76%)	-
- 2.4 mm plate	-	8/34 (23%)
- 2.0 mm plate	-	26/34 (77%)

^1^ Vitcone^®^ locking system; ^2^ Vi^®^ locking system.

**Table 3:** Twelve-week clinical and radiographic outcomes.

	Group A ^1^	Group B ^2^
- Lameness score = 0	52/59 (88%)	26/34 (76%)
- ROM reduction lateral approach distal fractures	0/34 (0%)	-
- ROM reduction lateral approach diaphyseal fractures	0/34 (0%)	-
- ROM reduction medial approach distal fractures	4/25 (16%)	5/34 (14.7%)
- ROM reduction medial approach diaphyseal fractures	-	-
- VAS *	4/4	4/4
- ICC	>0.75%	>0.75%
- 1-year telephone follow-up	47/59 (80%)	23/34 (67.6%)

^1^ Vitcone^®^ locking system; ^2^ Vi^®^ locking system; * VAS score: 0 = no healing; 1 = 1–25% healed; 2 = 26–50% healed; 3 = 51–75% healed; 4 = 76–100% healed.

**Table 4:** Postoperative implant management outcomes.

	Group A ^1^	Group B ^2^
- Gr implant	19/59 (32%)	22/34 (64%)
- En block plate removal	12/59 (20%)	-
- Staged plate dynamization	12/59 (20%)	12/34 (36%)
- Partial implant removal	16/59 (28%)	-
- Median removal timing (weeks)	14 (8–30)	28 (8–64)

^1^ Vitcone^®^ locking system; ^2^ Vi^®^ locking system.

**Table 5:** Major (implant failure and refracture after dynamization) and minor complications.

	Group A ^1^	Group B ^2^
- Total complications	5/59 (8.47%)	6/34 (17.64%)
- Implant failure (major)	1/59 (1.7%)	0/34 (0%)
- Refracture post dynamization (major)	4/40 (10%)	3/12 (25%)
- Minor complications	0/59 (0%)	3/34 (8.8%)

^1^ Vitcone^®^ locking system; ^2^ Vi^®^ locking system.

## Data Availability

The original contributions presented in the study are included in the article; further inquiries can be directed to the corresponding authors.
